# Ethical Challenges in Advanced Dementia: Role of Enteral Feeding Devices

**DOI:** 10.7759/cureus.95096

**Published:** 2025-10-21

**Authors:** Maria Luísa Olim, Sofia Osório Ferreira, Sara Santos, Luís Fernandes, Sérgio Gomes Ferreira, Gonçalo Carneiro, Lúcia Guedes

**Affiliations:** 1 Internal Medicine, Unidade Local de Saúde de Entre o Douro e Vouga, Santa Maria da Feira, PRT; 2 Palliative Care, Unidade Local de Saúde de Entre o Douro e Vouga, Santa Maria da Feira, PRT

**Keywords:** care ethics, dementia care, enteral nutrition, geriatrics, internal medicine and geriatrics

## Abstract

This review presents a critical analysis of enteral nutrition through feeding tubes in patients with advanced dementia, exploring medical, ethical, and sociofamilial aspects. The main goals include understanding the implications of artificial nutrition, considering human rights and medical ethics, and providing practical guidance for physicians and caregivers. We discuss different forms of enteral nutrition, such as nasogastric tubes and gastrostomies, highlighting specific indications and associated risks. The right to informed consent, treatment refusal, and advance directives is explored in detail. It is concluded that the decision to initiate artificial feeding should be a collaborative one, involving physicians, family members, and, when possible, the patient - always prioritizing the patient’s best interests. Continuous education, debunking myths, and flexibility in approach are essential. Additionally, respecting patient autonomy and supporting caregivers are fundamental to providing compassionate end-of-life care for patients with advanced dementia.

## Introduction and background

Dementia is a clinical syndrome characterized by global cognitive impairment with a decline in memory and other higher neurological functions, such as language, visuospatial perception, or executive function, which invariably results in functional dependence. It is currently estimated that 55 million people live with dementia worldwide, and this number is expected to increase to 139 million by 2050 [1].

The severity of dementia syndrome is determined based on evaluations of functional dependence, communication difficulties, and social interaction. It can be assessed using scales such as the Clinical Dementia Rating Scale or the Global Deterioration Scale [2-4]. 

The progression of dementia is highly variable among individuals and is often complicated by other comorbidities, making prognosis difficult to predict. The average survival has been estimated at 4.1 years. The survival time from the point of diagnosis in primary healthcare is estimated to be 6.7 years for patients aged 60 to 69, declining to 1.9 years when the diagnosis is made for patients aged 90 or older [5-7].

Feeding difficulties and weight loss are common features of dementia. In the early stages of the disease, feeding problems may be caused by the patient forgetting to eat or becoming disoriented during meals. There may also be changes in how food is perceived, including olfactory and taste disturbances that make food unappealing. In more advanced stages of dementia, swallowing difficulties (oropharyngeal dysphagia) may develop, leading to aspiration events and food refusal behaviors. The feeding disturbances present since the early stages of dementia become more prominent as the disease progresses. Throughout the progression of dementia, older adults may experience a range of nutritional challenges. In addition to dementia-specific factors, age-related comorbidities such as sarcopenia, dental impairments, mastication difficulties, and depressive symptoms can negatively influence dietary intake, ultimately increasing the risk of malnutrition [7-9].

In the preclinical and early stages of dementia, alterations in olfactory and gustatory perception are commonly reported. As the condition advances to mild or moderate stages, deficits in attention, executive functioning, and decision-making ability tend to become more evident. In the moderate to severe phases, dyspraxia and agnosia may emerge, frequently accompanied by behavioral disturbances. In the most advanced stage of the disease, severe oropharyngeal dysphagia and refusal to eat are often observed, further exacerbating nutritional decline [9].

Methodology

This review aimed to identify, analyze, and synthesize the scientific literature regarding the ethical principles involved in decisions to initiate or maintain enteral feeding in patients with dementia, with particular attention to dilemmas surrounding autonomy, beneficence, non-maleficence, and quality of life.

The guiding question was formulated according to the Preferred Reporting Items for Systematic Reviews and Meta-Analyses (PRISMA) 2020 framework [10], following the population-concept-context (PCC) structure: "What ethical principles are discussed in the literature regarding the use of enteral nutrition in patients with dementia?" (i) Population (P): Adults diagnosed with dementia. (ii) Concept (C): Enteral nutrition (nasogastric or percutaneous endoscopic gastrostomy feeding). (iii) Context (C): Ethical deliberation, clinical decision-making, palliative care, or end-of-life settings.

A comprehensive search was conducted between 1950 and 2025 in the following electronic databases: PubMed/MEDLINE, Scopus, Web of Science, and SciELO (to include literature in English, Portuguese, and Spanish). The search strategy combined medical subject headings (MeSH) and free-text terms, using Boolean operators (AND/OR). The main MeSH terms included: “Enteral Nutrition”, “Feeding Tubes”, “Dementia”, “Ethics”, “Ethical Theory”, “Decision Making”, “Palliative Care”, “Advance Directives”, “Quality of Life”, and “Aged”. Free-text terms such as “ethical dilemmas”, “tube feeding”, “artificial nutrition”, “moral principles”, and “end-of-life decisions” were also used.

Inclusion criteria were studies published between 1950 and 2025 in English, Portuguese, or Spanish. These included original research, systematic or narrative reviews, and theoretical or ethical analyses addressing ethical issues related to enteral feeding in patients with dementia. Exclusion criteria included studies focusing on parenteral nutrition, publications without an ethical component, letters to the editor, abstracts without full text, and pediatric studies.

The study selection followed PRISMA 2020 guidelines [10]. Search results were exported to the reference manager Mendeley (Mendeley Ltd., London, UK) for duplicate removal. Two independent reviewers screened the titles and abstracts, followed by full-text assessment of potentially eligible papers. Discrepancies were resolved through discussion or by a third reviewer.

Data were extracted using a standardized form including: author, year, country, study type, clinical context, main ethical issues identified, and key conclusions. A qualitative, descriptive, and thematic analysis was performed to identify the central ethical principles and recurring moral arguments across the studies. When applicable, methodological quality was assessed using the Critical Appraisal Skills Programme (CASP) checklist for qualitative studies, the A Measurement Tool to Assess Systematic Reviews (AMSTAR-2) tool for systematic reviews, and specific criteria for evaluating ethical and theoretical rigor in conceptual papers [11,12].

The main limitations of the review include heterogeneity of study designs, the scarcity of empirical research specifically focused on ethical decision-making, and potential language bias due to the exclusion of studies published in other languages.

The expected outcome of this review is to provide an integrative synthesis of the ethical principles guiding clinical decisions about enteral feeding in dementia, highlighting the balance between life prolongation and comfort, respect for autonomy and advance directives, moral responsibilities of healthcare professionals and families, and ethical recommendations for clinical practice.

## Review

Role of nutrition

Impact on Disease Morbimortality

Body weight loss in the elderly is often multifactorial and involves a combination of sarcopenia, metabolic and hormonal changes, inadequate nutrient intake, and the presence of chronic diseases, requiring a comprehensive medical approach for proper evaluation and treatment. The impacts of weight loss and malnutrition in older adults are well recognized, and it is important to consider a similar approach for individuals with dementia. Weight loss, which occurs at least partially as a consequence of decreased muscle mass (sarcopenia), results in functional decline and frailty, thereby increasing the risk of morbidity and mortality [7,8].

Impact on Disease Progression

The brain is a highly energy-demanding organ, requiring substantial metabolic resources to sustain normal functioning. Adequate nutrient supply is essential for maintaining cerebral integrity and metabolic activity. Pathophysiological processes such as vascular injury, oxidative stress, and inflammation - recognized as key contributors to dementia - can be influenced by specific dietary components. Severe deficiencies in micronutrients, including thiamine, folate, and vitamin B12, are strongly linked to cognitive dysfunction, while even milder insufficiencies are thought to play a role in cognitive decline [13-15].

Impact on Caregiver Burden

Most individuals with dementia live at home, where care is primarily provided by family members. The progressive loss of functional and cognitive abilities imposes physical, psychological, and financial strain on caregivers, and nutritional problems further intensify this burden. Caregivers often feel responsible for ensuring adequate intake, expressing concern about weight changes, dietary habits, and food refusal - behaviors that are difficult to interpret and manage. This bidirectional relationship, where nutritional difficulties heighten caregiver stress and caregiver overload worsens patient nutrition, has been confirmed in longitudinal studies, which show associations between caregiver burden, adverse eating behaviors, and weight loss in Alzheimer’s disease [16].

Thus, the use of certain nutritional interventions varies according to the patient's needs, the presence of advance directives, and the clinical team, preferably involving the caregivers' concerns. The beliefs of the patient and caregivers, as well as the wishes expressed in advance directives, may also vary according to the country, culture, and religious beliefs inherent to the individuals themselves [16,17].

Enteral nutrition

Although enteral nutrition technically encompasses the use of oral nutritional supplements, in the context of this review, the term will be restricted to nutrition delivered via feeding tubes. Enteral nutrition is generally the preferred method of artificial nutritional support for patients who are malnourished or at risk of malnutrition, provided gastrointestinal function is preserved, as it is considered more physiological, safer, and cost-effective than parenteral nutrition [17].

Percutaneous endoscopic gastrostomy (PEG) is an endoscopic procedure that establishes a gastrocutaneous fistula to permit direct delivery of nutrients to the stomach. PEG feeding represents a reliable and effective means of maintaining enteral nutrition in patients with significant swallowing impairment, offering several advantages over nasogastric tube (NGT) feeding. In clinical practice, PEG placement is usually recommended for patients expected to require enteral feeding for longer than four weeks, whether on a temporary basis due to reversible conditions or permanently in irreversible diseases, when life expectancy exceeds two months and cognitive function is relatively preserved. Conversely, NGT feeding is generally reserved for short-term support (up to 30 days), particularly in patients who maintain protective airway reflexes. Despite these general criteria, decisions to initiate tube feeding should be made on an individual basis, taking into account patient-specific needs, values, and family perspectives [18-20].

For individuals with mild to moderate dementia, nutritional support can often be provided through oral supplementation and, in acute and reversible circumstances, through temporary NGT placement, which may help improve nutritional status. In advanced dementia, however, oropharyngeal dysphagia and feeding difficulties are highly prevalent and frequently cited as justifications for tube feeding. Nevertheless, evidence increasingly indicates that in this population, artificial feeding does not confer benefits in terms of survival, nutritional improvement, or quality of life, leading many to regard the practice as both futile and ethically problematic [21]. Reflecting this evidence, professional societies such as the American Geriatrics Society (AGS) and the European Society for Clinical Nutrition and Metabolism (ESPEN) strongly advise against tube feeding in patients with advanced dementia, instead recommending careful assisted oral feeding as the more appropriate approach [19,20].

Despite established guidelines, the use of artificial nutritional support in the context of advanced dementia remains a widely employed intervention. It would be inappropriate to attribute this phenomenon solely to a specific factor, given that multiple factors may influence the use of enteral feeding devices in these individuals (Table 1) [22].

**Table 1:** Factors that may influence the use of enteral feeding devices in advanced dementia. Adapted from Ying (2015) [22], with permission.

Factors Affecting Enteral Feeding Devices	
Personal and family factors	Lack of knowledge or difficulty in coping with the poor prognosis of the disease
		Lack of awareness about the limited benefits and potential risks of tube feeding in advanced dementia
	Perception of inadequate nutritional intake, which can be influenced by religious and cultural factors
	The need for increased dedicated time for oral feeding
	Absence of advance directives concerning artificial nutrition and hydration
	Clinical factors	Lack of familiarity with techniques to manage thirst and hunger
Reluctance to engage in difficult conversations or assuming that another clinician will address the topic		
Fear of litigation	
Socioeconomic factors		Need for greater availability and additional human resources for oral feeding in patients with advanced dementia
	Financial incentives (e.g., institutions may benefit financially from caring for patients with feeding tubes)	

For many patients and their families, feeding and hydration hold significance beyond clinical necessity, often carrying strong personal, cultural, or religious meaning. Healthcare professionals may unintentionally reinforce the overuse of artificial nutrition and hydration devices. Misconceptions are also common among physicians, such as the erroneous assumption that aspiration pneumonia is a principal indication for tube feeding or the belief that enteral nutrition in advanced dementia improves nutritional outcomes or prolongs survival [23,24].

From an ethical standpoint, increasing attention has been given to respecting the values and wishes of patients and their caregivers. Families frequently view feeding and hydration as indispensable to life itself, which makes the issue particularly delicate when individuals cannot make decisions independently and lack advance directives or legal representation [25,26].

Overall, the body of evidence supporting current recommendations presents notable limitations. As a result, guidance discouraging artificial nutrition in advanced dementia may need to be revisited. In clinical practice, decisions should be individualized, grounded primarily in the patient’s previously expressed preferences, while also considering medical and nutritional requirements, potential discomfort, and the risk of complications.

Risks associated with artificial feeding and hydration in advanced dementia

In patients with advanced dementia, current recommendations advise against the use of long-term enteral feeding. As mentioned, there is no evidence that tube feeding is effective in improving quality of life, extending survival, or improving nutritional parameters. On the contrary, evidence suggests that it may even increase morbidity and mortality, as well as reduce quality of life. Furthermore, enteral nutrition in patients with severe dementia promotes the excessive use of restraints and is associated with increased discomfort, aspiration pneumonia, and worsening of urinary and fecal incontinence. Incontinence increases the risk of pressure ulcers and impairs the healing process [27-30].

The placement of an NGT is not without risks and complications, including the risk of obstruction and subsequent aspiration, gastroesophageal reflux, gastric mucosal irritation that may lead to gastrointestinal bleeding, and nasal injuries [18].

Endoscopic gastrostomy tube placement is an invasive procedure and can cause suffering, distress, and discomfort to patients. Associated complications can be categorized into minor or major, depending on their severity. These complications are predominantly observed in elderly patients with multiple comorbidities, compromised nutritional status, and a history of aspiration pneumonia or infections. Given the fragility of most patients undergoing endoscopic gastrostomy, the incidence of complications is non-negligible, ranging from 13% to 43% [18]. The complications described in the literature are related to the inherent risks of the procedure itself, such as wound infection, peristomal leakage, bleeding, internal organ injury, particularly intestinal perforation, and failure to heal the stoma. However, other complications, such as obstruction, migration, or extrusion of the tube, diarrhea, ileus, gastroesophageal reflux, aspiration pneumonia, necrotizing fasciitis, or buried bumper syndrome, are also described. A low mortality rate is also noted in association with the technique (0-2%), although this rate significantly increases at 30 days (6.7-26%), especially in patients with cardiovascular comorbidities [18].

Enteral feeding can be particularly problematic in patients with dementia, who may not recognize the device. Failure to recognize the feeding tube can lead to attempts at removal. To reduce this risk, as previously mentioned, patients may require physical (e.g., using gloves) or chemical (e.g., through sedation) restraints [18,27-30].Table 2 presents some of the risks and harms associated with artificial nutrition in advanced dementia [22].

**Table 2:** Risks and potential harms associated with the use of enteral feeding devices in advanced dementia. Adapted from Ying (2015) [22], with permission.

Risks and Potential Harms
Pain and other complications (e.g., infection, bleeding) directly associated with the placement of the feeding tube
Increased risk of aspiration
Increased risk of pressure ulcers
Gastrointestinal symptoms resulting from tube feeding (e.g., diarrhea, constipation, gastroesophageal reflux)
Use of physical restraints to prevent the patient from removing the feeding tube
Fluid overload promoting the development of pulmonary or peripheral edema, as well as an increase in secretions in the upper airways
Increased perception of hunger

Informed consent and the right of refusal

Informed Consent

Informed consent is defined as the explicit and voluntary authorization provided by a patient prior to undergoing a medical intervention. This authorization requires that the individual be fully informed about the nature of the procedure, the techniques involved, its purpose, and the anticipated benefits and risks. As emphasized by international bioethical guidelines, no clinical act should be performed without the patient’s free and informed agreement, which constitutes both an ethical obligation and a legal duty for healthcare professionals [25,26]. The regulatory frameworks governing the circumstances under which informed consent is required, as well as the manner in which it is obtained, differ considerably across jurisdictions.

In the Portuguese context, consent may be expressed in writing, verbally, or through any other unequivocal manifestation of will. The form of consent must be proportionate to the degree of risk associated with the intervention. Several modalities are recognized: tacit or implicit consent, when acceptance is evident from the patient’s conduct; presumed consent, which applies in emergency settings when the patient cannot communicate their preferences and no prior refusal has been registered; and third-party consent, in which decisions are delegated to a legal representative or, in specific cases, to judicial authorities, particularly when patients lack decisional capacity, as may occur in advanced stages of dementia [31,32].

End-of-life decision-making often generates substantial psychosocial distress for families and caregivers. Accordingly, health professionals are urged to avoid placing the entire burden of such decisions on relatives and instead to promote shared decision-making approaches. In situations where a legal representative refuses to authorize treatment but the physician judges that withholding intervention would seriously endanger the patient, the matter may be referred to the competent judicial authority [25,26].

The Universal Declaration of Human Rights underscores that informed consent must always be voluntary and free from coercion. The ability to provide valid consent depends on decisional capacity, which cannot be assumed to be lost solely on the basis of a dementia diagnosis [33]. Thus, the assessment of mental capacity is a critical component in the care of individuals with dementia or altered states of consciousness.

Advance Directives and Treatment Refusal

Advance care planning is increasingly recognized as an essential component of patient-centered healthcare, ensuring that future clinical decisions align with individual values and preferences. A central mechanism in this process is the advance directive, which allows individuals to document choices about medical treatment should they later lose the capacity to decide for themselves. Such directives reflect the broader principle that competent adults possess the legal and moral right to refuse medical treatment, even when such refusal may result in the shortening of life expectancy [26,34,35].

The refusal of treatment, however, presents a series of ethical and legal challenges. Central among these are questions of whether the refusal is genuinely informed, whether the individual possesses sufficient decision-making capacity, and under what circumstances, if any, third parties such as family members or legal authorities might legitimately override the patient’s expressed wishes [36].

When valid and applicable, advance refusals are regarded as having the same normative authority as contemporaneous refusals made by patients with intact capacity [36]. Despite this, clinicians may encounter ethical tensions when directives appear to conflict with clinical judgment. For example, dilemmas arise in cases where an intervention, such as short-term enteral feeding, is intended to be temporary and potentially beneficial, yet the advance directive does not explicitly distinguish between rejecting burdensome life-sustaining measures and refusing time-limited treatments with a favorable prognosis [26].

Within medical ethics, it is widely accepted that there is no substantive moral difference between a patient’s decision to decline treatment from the outset and a decision to withdraw that treatment once it has been initiated [36]. This principle underscores the importance of respecting patient autonomy while simultaneously recognizing the moral complexity that healthcare professionals may face when applying advance directives in practice.

Ethical considerations of enteral nutrition

Patients who experience difficulties with oral feeding present complex legal, moral, and ethical dilemmas for healthcare professionals. Such difficulties can occur in individuals with physical impairments, neurological conditions, or cognitive decline, and may arise at any stage of life, including during the final phases of illness. As with all clinical interventions, the management of feeding disorders must be preceded by a thorough evaluation and the informed consent of the patient or, where applicable, their legal representative. Depending on the severity of the swallowing disorder, therapeutic strategies may involve dietary adjustments, texture modification of food, or, in more severe cases, the initiation of artificial nutrition through nasogastric intubation or percutaneous gastrostomy [26].

The practice of clinical ethics, which is rooted in bioethical theory, provides the framework through which these challenges are addressed in modern healthcare systems. Within the paradigm of patient-centered care, the guiding aim is to ensure that interventions are consistent with the patient’s values and preferences. To support this aim, the integration of evidence-based medicine is recommended, as it promotes interventions that enhance clinical effectiveness, reduce the risk of harm, and facilitate the fair and equitable allocation of healthcare resources [36].

Bioethical Considerations in Dysphagia Care

Clinical decision-making is commonly guided by four central ethical principles: beneficence, non-maleficence, respect for autonomy, and justice. Beneficence emphasizes the clinician’s duty to act in ways that promote the well-being of the patient, whereas non-maleficence underscores the obligation to avoid causing harm [36]. In the management of swallowing disorders, these principles translate into offering effective therapeutic options for dysphagia. Nonetheless, in circumstances where the potential outcomes are uncertain and the risk of harm outweighs the benefits, the decision to refrain from intervention may be ethically and morally defensible [26,36].

Autonomy highlights the importance of recognizing and upholding patients’ values, preferences, and healthcare-related goals. Respecting this principle includes acknowledging an individual’s right to decline or discontinue treatment, even when such interventions could extend life, irrespective of whether the patient is approaching the end of life. The principle of justice requires that all patients be treated fairly and without bias, regardless of socioeconomic background, ethnicity, religion, or other personal characteristics. In clinical practice, this necessitates that healthcare professionals cultivate awareness and sensitivity to the plurality of perspectives held by patients and their families [36].

The Right to Adequate Nutrition

It is worth highlighting that two provisions of the European Convention on Human Rights (1950) are particularly relevant to the use of artificial nutrition in individuals with advanced dementia. Article 2, which enshrines the right to life, affirms that “everyone’s right to life shall be protected by law.” Article 3 prohibits torture and explicitly states that “no one shall be subjected to torture or to inhuman or degrading treatment or punishment” [33]. Within this framework, the practice of administering enteral feeding against a patient’s will may be interpreted as a form of assault. Furthermore, initiating or continuing a treatment such as artificial nutrition that offers no tangible clinical benefit is ethically problematic, as it risks inflicting greater harm than good on the patient.

Terminal Dehydration

In certain acute and reversible clinical conditions, such as hypercalcemia, fever, or diarrhea, hydration support may be required. Among patients with advanced dementia, difficulties such as dysphagia or impaired perception of thirst can compromise the ability to maintain adequate fluid intake, making parenteral supplementation necessary [37]. Intravenous hydration is typically considered the standard approach when venous access is already established for other treatments. However, when adherence is poor, peripheral access is difficult, central venous access is not feasible, or the patient is being cared for at home or in a long-term facility, subcutaneous infusion offers a practical alternative. The administration of fluids into the subcutaneous tissue - hypodermoclysis - represents a simple, safe, and effective technique for managing mild to moderate dehydration, particularly in individuals with cognitive impairment. This method is associated with minimal discomfort during placement and maintenance, and patients are generally less inclined to tamper with the access site. Available evidence indicates that hypodermoclysis provides hydration outcomes comparable to intravenous fluid therapy [38].

At the end of life, decisions regarding the use of enteral or parenteral hydration must weigh the potential benefits against the burdens, including pain and discomfort. It is well established that thirst can often be effectively alleviated through simple local measures [39,40]. Education is essential to dispel persistent myths surrounding artificial nutrition in advanced dementia, particularly caregiver concerns regarding hunger and thirst. Evidence suggests that intravenous hydration does not significantly relieve thirst or dry mouth, and in patients with advanced disease, enteral feeding may even intensify sensations of hunger. Instead, symptoms at this stage are best managed with small quantities of food or fluids, the use of artificial saliva, and careful oral hygiene [38].

The Dilemma of Intervention

The central ethical challenges in dementia care concern who should receive treatment, when interventions are appropriate, and how they ought to be delivered. As several scholars have emphasized, there is no universally applicable solution to these dilemmas. When formulating a treatment plan, two dimensions require careful consideration: the patient’s individual values and expectations, and the balance between potential burdens such as pain, discomfort, or distress and potential benefits including recovery, nutritional status, quality of life, and overall well-being [25,26]. Predicting long-term outcomes in dementia is notoriously difficult, while the immediate complications and discomfort of interventions are often more visible. Furthermore, what may be regarded as a positive outcome for one patient might be deemed unacceptable by another.

These dilemmas are particularly acute in cases where the prognosis is uncertain. Should enteral nutrition be initiated? Can it be introduced provisionally and later discontinued? Should artificial feeding be considered a medical treatment or an inalienable right? Within the framework of common law, enteral nutrition is legally classified as a medical intervention and may therefore be prescribed, initiated, and subsequently withdrawn when clinically or ethically justified [25,26,36].

In advanced dementia, where decision-making capacity is significantly impaired, additional ethical questions arise in situations of food refusal. Forced feeding may constitute a form of assault, and the use of physical restraints requires critical evaluation. Is it ethically defensible to restrain a patient solely to prevent the removal of feeding tubes? What ultimate goals are being pursued in doing so? In many cases involving irreversible decline, final decisions are not fundamentally altered but rather delayed, prolonging uncertainty for both patients and caregivers [39,40].

## Conclusions

Enteral nutrition in individuals with advanced dementia remains a subject of considerable debate, and this practice continues to raise difficult questions. Decisions regarding the initiation of tube feeding should be made jointly, with input from the treating physician, the patient’s relatives, and, whenever feasible, the patient. Ethical considerations play a central role in this process, making it essential to acknowledge and address the worries of families and caregivers before balancing possible advantages against the likelihood of harm. Ideally, the choices of a legal proxy ought to align with the patient’s prior wishes, or, if these are unknown, be guided by what would best serve the patient’s interests.

When artificial feeding is initiated, physicians should set realistic expectations while remaining flexible in their approach. Caregivers need guidance on signs that may require adjustments or removal of the device, such as shortness of breath from pulmonary edema, diarrhea, pressure ulcers, or attempts by the patient to remove the tube. Early conversations about the limited evidence for enteral nutrition in late-stage dementia are essential, but this does not mean the intervention is never appropriate. Systematically excluding tube feeding for all patients risks undermining the ability of healthcare professionals to provide individualized, patient-centered care.
